# Rab GTPases, tethers, and SNAREs work together to regulate *Arabidopsis* cell plate formation

**DOI:** 10.3389/fpls.2023.1120841

**Published:** 2023-02-10

**Authors:** Yumei Shi, Changxin Luo, Yun Xiang, Dong Qian

**Affiliations:** Ministry of Education (MOE) Key Laboratory of Cell Activities and Stress Adaptations, School of Life Sciences, Lanzhou University, Lanzhou, China

**Keywords:** *Arabidopsis*, vesicle trafficking, Rab GTPases, tethers, SNAREs, cytokinesis, cell plate

## Abstract

Cell plates are transient structures formed by the fusion of vesicles at the center of the dividing plane; furthermore, these are precursors to new cell walls and are essential for cytokinesis. Cell plate formation requires a highly coordinated process of cytoskeletal rearrangement, vesicle accumulation and fusion, and membrane maturation. Tethering factors have been shown to interact with the Ras superfamily of small GTP binding proteins (Rab GTPases) and soluble N-ethylmaleimide-sensitive factor attachment protein receptors (SNAREs), which are essential for cell plate formation during cytokinesis and are fundamental for maintaining normal plant growth and development. In *Arabidopsis thaliana*, members of the Rab GTPases, tethers, and SNAREs are localized in cell plates, and mutations in the genes encoding these proteins result in typical cytokinesis-defective phenotypes, such as the formation of abnormal cell plates, multinucleated cells, and incomplete cell walls. This review highlights recent findings on vesicle trafficking during cell plate formation mediated by Rab GTPases, tethers, and SNAREs.

## 1 Introduction

Cell division is fundamental to plant growth, development, and reproduction, including the processes of DNA replication, nuclear division, and cytokinesis (Tulin and Cross, 2014). Cytokinesis is the final step in cell division, which involves the process of separating a mother cell into two daughter cells by forming a new compartment between two newly formed daughter nuclei. This is a highly coordinated spatiotemporal event that involves specialized rearrangements of the cytoskeleton during cell division and a series of vesicle transport activities (Müller, 2019; Yi and Goshima, 2022). During cytokinesis, the aggregation and alignment of microtubules forms the phragmoplast, which promotes the orderly delivery of vesicles at the plane of cell division; furthermore, the fusion and fission of aggregated vesicles in the center of dividing cells promotes early cell plate formation (Euteneuer and McIntosh, 1980; Lee et al., 2001; Jürgens, 2005).

**Figure 1:**
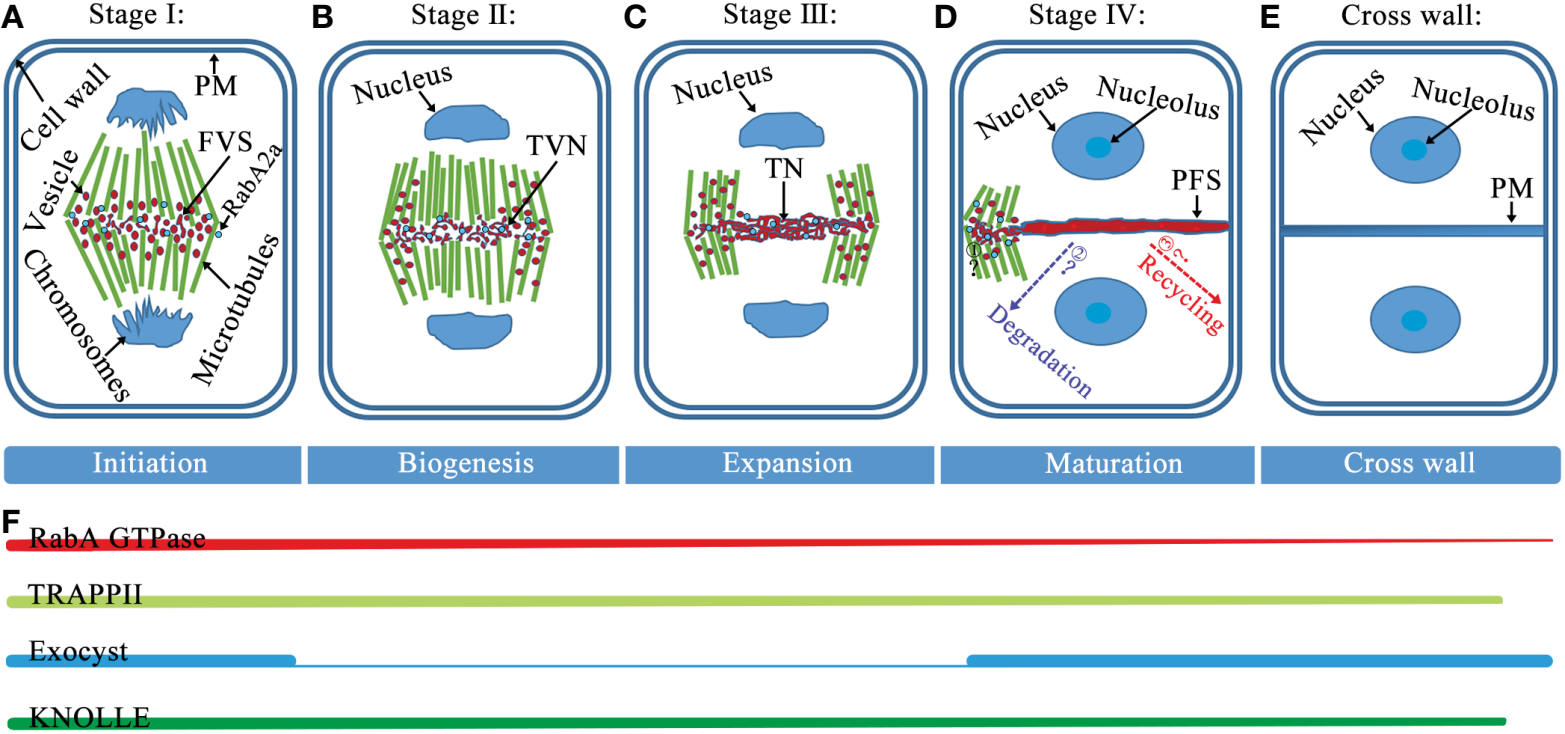
Model of cell plate formation stages and spatiotemporal distribution of Rab GTPases, tethers, and SNAREs. **(A)** Stage I, at this fusion of vesicles stage (FVS), during the initial fusion and fission of the bladder, the dumbbell structure forms. **(B)** Stage II, vesicles undergo fusion, fission, and conformational changes to form a tubulo-vesicular network (TVN). **(C)** Stage III, the TVN gradually merges into a tubular network (TN). **(D)** Stage IV, as the cell plate continues to smoothen and expand, the formation of a planar fenestrated sheet (PFS). The cell plate extends fusion tube connecting to the cell plate fusion site with PM and fuses with PM (Step1), and the black question mark indicates an unknown mechanism. After the cell plate is anchored to PM, the proteins diffused from the cell plate would be recycled and/or degraded (Step2 and 3), and dashed arrows with question marks indicate where these proteins are likely to go. **(E)** At the end of cytokinesis, the cell plate enters maturation when the new primary cross wall and daughter cells separate. **(F)** The association of RabA GTPase with the membrane provides cargo for cell plate formation and remains present throughout cell plate formation. Both TRAPPII and exocyst complexes are present at the onset of cytokinesis. Thereafter, the TRAPPII complex consistently marks the cell plate from cytoplasmic division and it is required for its biogenesis, while the outer capsule is primarily required for the maturation of the cell plate. Throughout the cell plate formation phase, SNARE-dependent membrane vesicles fuse to form the cell plate. Abbreviations: PM, plasma membrane; FVS, fusion of vesicles stage; TVN, tubulo-vesicular network; TN, tubular network; and PFS, planar fenestrated sheet.

The formation of the cell plate goes through the following four distinct stages (**Figure 1**): (i) The Golgi-derived vesicles are guided to the cell division plane by the phragmoplast, and vesicles aggregate and fuse to form dumbbell structures. (ii) The initial collection of fused tubes at the center of the segmentation plane undergo a series of morphological changes, resulting in a tubulo-vesicular network, depolymerization of the microtubules underlying the tubulo-vesicle network, and stabilization of the microtubules adjacent to the edge of the fusion channel (Nishihama and Machida, 2001; Seguí-Simarro et al., 2004). (iii) Gradual merging into a tubular network form, which is a membrane morphology that subsequently forms into a smoother structure largely through network expansion. (iv) Formation of a fenestrated sheet that fuses with the parental plasma membrane (PM) (Samuels et al., 1995; Sinclair et al., 2022). This process involves various actions such as closing the plate fenestrae, adding pectin and xyloglucan, removing excess membranes, and replacing callose with cellulose. Eventually, the cell plate fuses with the mother cell wall, and the process ends with a transition to an entirely new lateral wall that separates the daughter cells (Samuels et al., 1995; Seguí-Simarro et al., 2004; Baluska et al., 2005).

**Figure 2:**
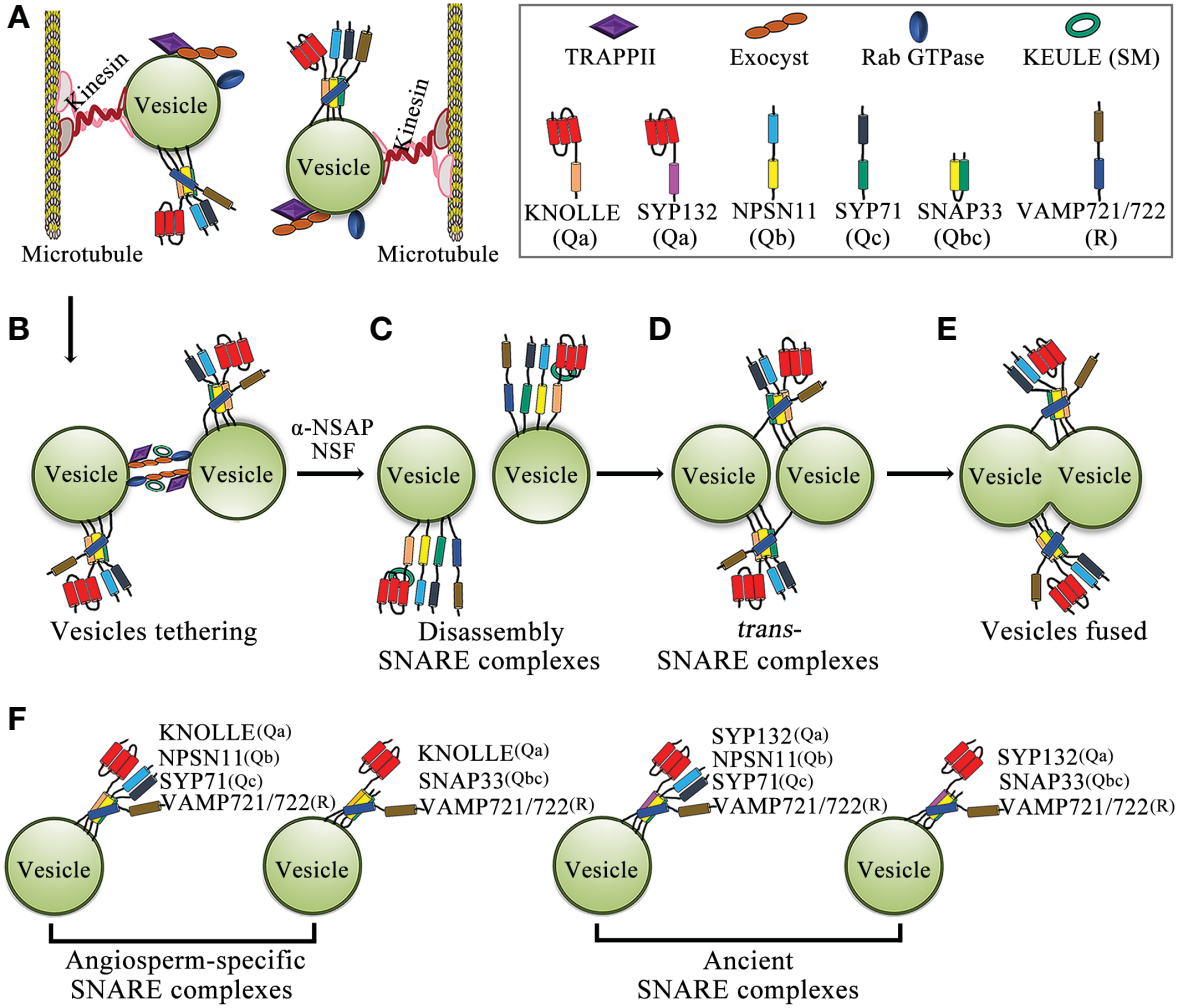
Schematic model of membrane-vesicle fusion during cytokinesis. **(A)** Vesicles carry Rab GTPase, two types of tethering complexes, TRAPPII and exocyst, and the KNOLLE-containing *cis*-SNARE complex through kinesin transport along microtubules to the plane of cell division. **(B)** Rab GTPases promote the tethering of two adjacent vesicles by tethering complexes (TRAPPII and exocyst). **(C)** The *cis*-SNARE complex is disassembled by NSF-ATPase and α-SNAP, and the Qa-SNARE KNOLLE interacts with the Sec/Munc18 protein KEULE to keep the KNOLLE in an open conformation. **(D)** KNOLLE interacts with SNARE partners of adjacent vesicles to form *trans*-SNARE complexes. **(E)** Two adjacent vesicles fused together. **(F)** Model of the SNARE complex in cytokinesis. The cytokinesis-specific Qa-SNARE KNOLLE forms two types of SNARE complexes. In addition, the evolutionarily ancient Qa-SNARE SYP132 forms two types of SNARE complexes. Abbreviations: SM, Sec1p/Munc18; α-SNAP, α-soluble NSF attachment protein; and NSF, n-ethylmaleimide-sensitive factor.

**Figure 3:**
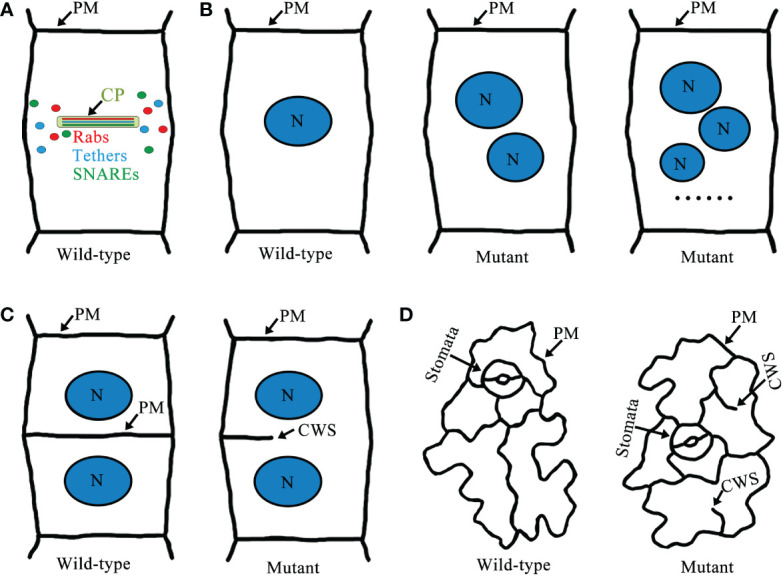
A schematic model of Rab GTPases, tethers, SNAREs cell plate localization and cytokinesis-defective mutants in *Arabidopsis*. **(A)** Localization of Rab GTPases, tethers, and SNAREs in the cell plate. **(B)** Wild-type root epidermal cells of *Arabidopsis* have normal cytokinesis and only one nucleus per cell. In cytokinesis-defective mutants there are two or more nuclei per cell. **(C)** Wild-type root epidermal cells have intact cell walls, but defects in cell plate formation in cytokinesis-defective mutants lead to the formation of cell wall stubs. **(D)** Wild-type cotyledon epidermal cells are intact cells with clear outlines. However, cytokinesis-defective mutants often have cell wall stubs. Abbreviations: CP, cell plate; N, nucleus; PM, plasma membrane; and CWS, cell wall stubs.

During cell plate biogenesis, cytokinetic vesicles deliver cargo and contribute membrane material. Cytokinetic vesicles are primarily derived from the Golgi/*trans*-Golgi network (TGN) and are contributed by endosomal populations. ARF guanine exchange factors (ARF GEFs) BIG1-4 assist in the transport of newly synthesized proteins and endocytic products to the formed cell plate (Seguí-Simarro et al., 2004; Richter et al., 2014). Intracellular membrane fusion generally depends on Rab GTPases, tethering factors, and SNARE proteins (Jahn et al., 2003; Jahn and Scheller, 2006; Stenmark, 2009; Hong and Lev, 2014). Rab GTPases are master regulators of membrane trafficking, regulating the transport of vesicles during cell plate formation (Davis et al., 2016; Minamino and Ueda, 2019). Activation of Rab GTPases by GEFs promotes recruitment of tethering factors to the membranes (Stenmark, 2009). Tethering proteins provide specificity for targeting, and vesicle tethering initiates SNARE-dependent fusion of membrane vesicles to form cell plates (Yu and Hughson, 2010) (**Figure 2**). Some Rab GTPases, tethers, and SNAREs are localized to the cell plate during cytokinesis, and some of these mutations lead to typical cytokinesis-defective phenotypes, such as the formation of abnormal cell plates, binucleated or multinucleated cells, and cell wall stubs (Chow et al., 2008; Jaber et al., 2010; Qi et al., 2011; Zhang et al., 2011) (**Figure 3**).

## 2 The Rab family of small GTPase proteins are involved in plant cell plate formation

Rab GTPases are members of the Ras-like small GTP-binding protein superfamily, which are guanine nucleotide-binding proteins that act as molecular switches that can alternate between the following two conformations: the inactive form (GDP-bound) and the active form (GTP-bound). The conversion of Rab GTPases from a GDP-bound to a GTP-bound form requires a GEF (Cui et al., 2014; Rosquete et al., 2019). The Rab GTPase plays an important role in various forms of membrane transport, by activating and/or recruiting various membrane traffic regulators (also known as Rab effector proteins) (Prekeris, 2003; Sohn et al., 2003; Hutagalung and Novick, 2011). Rab GTPases are involved in the regulation of multiple cellular processes, including endosome organization, PM recycling, phagocytosis, cytokinesis and so on (Pereira-Leal and Seabra, 2001; Woollard and Moore, 2008). Cytokinesis requires the activity of Rab GTPase to regulate vesicle-mediated material contributions to the developing cell plate (Chow et al., 2008). The *Arabidopsis* genome encodes at least 57 members of the Rab GTPases, which are grouped into eight subfamilies (RabA GTPase to RabH GTPase) (Vernoud et al., 2003; Woollard and Moore, 2008). Four subfamilies of Rab GTPases (RabA, RabE, RabF and RabH) are involved in the formation of the cell plate during cytokinesis (**Table 1**).

**Table 1:** Characteristics of Rab GTPases located in cell plates.

Type	Gene	AGI Gene	Localization	Function	Reference
RabA	RabA1b/ BET5	AT1g16920	TGN/EE, PM, CP	Secretory pathway from TGN to PM; exocytic trafficking; recycling pathways.	Feraru et al., 2012 ; Asaoka et al., 2013
	RabA1c	At5g45750	TGN/EE, CP	Involved in cytokinesis; the polar secretion and circulation of PM proteins.	Qi et al., 2011; Qi and Zheng, 2013
	RabA1d	At4g18800	TGN/EE, CP	Cell plate formation and polarized cell expansion of root hairs; regulates vesicular trafficking at TGN.	Takáč et al., 2012; Berson et al., 2014
	RabA1e	At4g18430	E, RE, CP	Vesicle-mediated cargo delivery during cytokinesis and root hair elongation.	Geldner et al., 2009; Berson et al., 2014; Davis et al., 2016
	RabA2a	At1g09630	TGN/EE, CP	Involved in cytokinesis; vesicle secretion regulates vesicle trafficking from the TGN to the PM; regulation of K^+^ homeostasis.	Chow et al., 2008; Park et al., 2014; Davis et al., 2016; Pang et al., 2022
	RabA2b	At1g07410	TGN/EE, PM, CP	Mediated PM trafficking to improve drought tolerance.	Chow et al., 2008; Ambastha et al., 2021
	RabA2c	At3g46830	TGN/EE, CP	Vesicle secretion and vesicle trafficking.	Chow et al., 2008
	RabA2d	At5g59150	TGN/EE, CP	Vesicle secretion and vesicle trafficking.	Chow et al., 2008
	RabA3	At1g01200	TGN/EE, CP	Vesicle secretion and vesicle trafficking.	Chow et al., 2008
	RabA5c	At2g43130	TGN/EE, CP	Involved in cytokinesis; regulates the specification of geometric edges in directional cell growth lateral roots; specifies a secretory pathway from the TGN/EE to the PM.	Rahni and Birnbaum, 2016; Kirchhelle et al., 2016; Kirchhelle et al., 2019; Elliott et al., 2020
RabE	RabE1c	At3g46060	Golgi, PM, CP	Post-Golgi trafficking to the PM; involved in the degradation of the peroxisomal protein receptor peroxin 7.	Speth et al., 2009; Ahn et al., 2013; Cui et al., 2013; Mayers et al., 2017
	RabE1d	At5g03520	Golgi, PM, CP	Response to pathogen; secretory pathways from the Golgi to the PM.	Zheng et al., 2005; Chow et al., 2008; Speth et al., 2009
RabF	RabF1/ ARA6	At3g54840	PM, MVEs, RE, CP	Trafficking pathway from endosomes to the PM; may be involved in recycling and degradation.	Dhonukshe et al., 2006; Bottanelli et al., 2011; Ebine et al., 2011; Ebine et al., 2012; Inada et al., 2017
	RabF2b/ ARA7	At4g19640	LE, PVC, TGN/EE, CP	Involved in cytokinesis; endocytosis; vesicle transport between the PVC and the vacuole.	Dhonukshe et al., 2006; Jia et al., 2013; Ito et al., 2016
RabH	RabH1b	At2g44610	Golgi, TGN/EE, CP	Influences cell elongation/growth and cellulose biosynthesis in hypocotyl growth; regulating the transport of cellulose synthase proteins between the Golgi apparatus and PM.	Chow et al., 2008; Johansen et al., 2009; He et al., 2018; Jia et al., 2018

E, endosome; TGN, trans-Golgi network; EE, early endosome; LE, late endosome; RE, recycling endosome; PVC, pre-vacuolar compartment; PM, plasma membrane; MVEs, multivesicular endosomes; and CP, cell plate.

Ten RabA GTPases (RabA1b, RabA1c, RabA1d, RabA1e, RabA2a, RabA2b, RabA2c, RabA2d, RabA3, and RabA5c) are localized to the cell plate (**Table 1**). The gene BEX5 encodes RabA1b, which localizes to the TGN/EE, PM, and cell plates, and it functions in protein trafficking in *Arabidopsis* roots, presumably by regulating vesicle formation, budding, and trafficking from the TGN/EE to the PM/cell wall (Geldner et al., 2009; Feraru et al., 2012; Asaoka et al., 2013). *Bex5* mutants display the following defects: increased protein accumulation in abnormal trafficking inhibitor brefeldin A (BFA) compartments, abnormal endosomes, and defects in both exocytosis and transcytosis of PM proteins (Feraru et al., 2012). During cell division, RabA1c is relocated to the cell plate, and this process can be interrupted by the chemical compound endosidin 1 (ES1). In addition, RabA1c defines a group of TGNs that are related to VHA-a1-tagged TGN but only partially overlap with them (Qi and Zheng, 2013). RabA1c (S27N) and RabA1c (Q72L), which are dominant inhibitory mutants, are impaired in root growth and show severe cytokinesis defects (Qi and Zheng, 2013). In addition, root growth and cytokinesis in root cells of *raba1a/b/c* triple mutant seedlings are sensitive to low levels of ES1 (Kotzer et al., 2004; Lee et al., 2004; Qi and Zheng, 2013). RabA1d is localized at the TGN/EE and cell plates and is involved in vesicle trafficking and cell plate formation. The accumulation pattern of RabA1d is consistent with regions of active vesicle fusion during cell plate formation and cell growth, which suggests that it plays an important role in cell plate formation and membrane/cargo trafficking for membrane recycling (Takáč et al., 2012; Berson et al., 2014). RabA1e appears on the cell plate in cytokinesis and it may mediate vesicle transport during cytokinesis. In early-stage cell plates, YFP-RabA1e and YFP-RabA2a were consistently localized to a disk-shaped structure at the center of the dividing cell. In late-stage cell plates, the localization patterns of the two proteins were different, in which YFP-RabA2a were mainly localized to ring-shaped structures across the cell division plane, whereas YFP-RabA1e were mainly localized to both ring-shaped structures and disk-shaped structures. In addition, in late-stage cell plates, differences between YFP-RabA2a and YFP-RabA1e were more pronounced after treatment of cytokinesis inhibitor endosidin 7. RabA1e and RabA2a exhibit different subcellular behaviors, which implies that their localization and transport functions may involve different cellular components (Chow et al., 2008; Berson et al., 2014; Davis et al., 2016). In *Arabidopsis*, the small GTPases RabA2 (RabA2a, RabA2b, RabA2c, and RabA2d) and RabA3 are preferentially localized to the leading edge of the cell plate, implying that RabA2 and RabA3 play a role in the delivery and incorporation of novel substances into the assembled cell plate (Chow et al., 2008; Park et al., 2014; Mayers et al., 2017). Inducible expression of dominant inhibitory mutants of RabA2a (S26N), RabA2a (Q71L), and RabA2a (N125I) results in severely disrupted cell division patterns, binucleate and multinucleate cells, and significant inhibition of cytokinesis (Söllner et al., 2002; Chow et al., 2008). These results demonstrate that RabA2a is required for cytokinesis and transport to the cell plate *via* the Golgi and TGN, possibly by regulating secretion or endocytosis associated with cell plate development. RabA5c accumulates in unique vesicles and sometimes in the TGN, resides at the cell plate, and promotes cytokinesis (Kirchhelle et al., 2016; Kirchhelle et al., 2019; Elliott et al., 2020). Inducible expression of RabA5c (N25I) resulted in severe restriction of root growth, grossly abnormal cell geometries, and incomplete and misaligned cytokinesis in lateral roots (Kirchhelle et al., 2016).

In addition to RabA GTPases, there are five other subfamilies of Rab GTPases located in the cell plate of dividing cells, including two RabE GTPases (RabE1c and RabE1d), two RabF GTPases (RabF1 and RabF2b), and one RabH GTPase (RabH1b) (**Table 1**). Five members of the RabE subfamily (RabE1a to RabE1e) are believed to regulate post-Golgi trafficking to the PM, and live cell imaging shows that RabE1d and RabE1c localize to the Golgi apparatus, PM, and cell plate of dividing cells (Vernoud et al., 2003; Zheng et al., 2005; Chow et al., 2008; Speth et al., 2009). RabE1 interacts with the stomatal cytokinesis defect (SCD) complex, a multiprotein complex that in turn interacts with exocyst components to jointly promote secretion and endocytosis during cytokinesis; furthermore, overexpression of RabE1c rescues the growth and guard cell cytokinesis phenotypes of the temperature-sensitive mutant *scd1-1* (Mayers et al., 2017). In fixed *Arabidopsis* roots, RabF1 (Ara6) and RabF2b (Ara7) are localized to the cell plate and they are involved in the formation of cell plates during cytokinesis. *Arabidopsis* seedlings expressing dominant-negative RabF2b (Ara7 S24N) show stunted growth, root tip structure disorder, abnormal cytokinesis with multinucleated cells and incomplete cell walls (Dhonukshe et al., 2006). Interestingly, weak fluorescence is generally observed for YFP: RabH1b on the cell plate in addition to the Golgi localization signal, but the intensity of YFP: RabH1b signaling on the cell plate never exceeds the intensity in the same Golgi stack cells (Chow et al., 2008; He et al., 2018; Renna et al., 2018). These findings show that Rab GTPase plays an important role in vesicle trafficking during cell plate formation.

## 3 Tethering complexes involved in cell plate formation

Tethers refer to the initial contact between the donor and acceptor membranes, which is a highly selective transport process that facilitates vesicle docking and fusion. The initial connection between the carrier vesicle and its target membrane requires tethers, but not all putative tethers can bind the vesicle (Cai et al., 2007). Tethering factors fall into the following two main categories: long putative coiled-coil proteins and multisubunit tethering complexes (Cai et al., 2007; Koumandou et al., 2007; Ravikumar et al., 2017). Tethering complexes act by capturing vesicles and holding them in the vicinity of the target membrane, thereby they play an important role in cell plate assembly (Vukašinović and Žárský, 2016). Of these tethering factors, two important classes of tethering complexes, TRAPPII and exocysts, are required for plant cytokinesis (Rybak et al., 2014). In *Arabidopsis*, the TRAPPII complex consists of ten subunits, including the previously discovered TRAPPII subunits (Bet3, Bet5, Trs20, Trs23, Trs31, Trs33, Tca17, Trs120, and Trs130) and the recently reported plant-specific component TRAPP-interacting plant protein (TRIPP) (Zhang et al., 2018; Garcia et al., 2020). The *Arabidopsis* TRAPPII complex was discovered by screening cytokinesis-defective mutants and it is required for cell plate biogenesis (Jaber et al., 2010; Rybak et al., 2014). The exocyst is an evolutionarily conserved tethered complex consisting of eight subunits (Sec3, Sec5, Sec6, Sec8, Sec10, Sec15, Exo70, and Exo84). The exocyst and other regulatory proteins tether secretory vesicles to the cell membrane prior to membrane fusion, and the exocyst is necessary for the maturation of the cell plate during cytokinesis (He and Guo, 2009; Heider and Munson, 2012; Rybak et al., 2014). Two tethering complexes, TRAPPII and the exocyst, physically interact with each other and coordinate the spatiotemporal regulation of cell plate initiation (Rybak et al., 2014; Müller and Jürgens, 2016). During the initiation and maturation of the cell plate, TRAPPII colocalizes with exocysts and persists there during cell plate assembly. Switching between these tethering complexes is associated with changes in the membrane properties and mediates the biogenesis of the cell plate through distinct stages (**Figure 1**) (Rybak et al., 2014).

Four TRAPPII subunits (TRS120/VAN4, TRS130/CLUB, TPIPP and TRS33) are essential for cell plate formation (**Table 2**). TRS120 and TRS130 are localized in the TGN/EE and cell plate, and they are required for cell plate biogenesis (Ravikumar et al., 2017, 2018). Mutations in TRS120 or TRS130 result in a lethal and typical cytoplasmic defect in seedlings, including cell wall stubs, multinucleate cells, and incomplete connective walls. In addition, in both mutants, vesicles aggregate at the division plane but fail to assemble into the cell plate (Jaber et al., 2010; Thellmann et al., 2010; Qi et al., 2011; Ravikumar et al., 2017; Ravikumar et al., 2018). Interestingly, organization and trafficking of the endoplasmic reticulum (ER)-Golgi interface are normal in *trs120* and *trs130* mutants; however, trafficking from the post-Golgi to the cell plate and cell wall, but not to the vacuole, is impaired (Qi et al., 2011). Recently, TRIPP was found to be a plant-specific member of the highly conserved TRAPPII complex, which is localized to TGN/EE in interphase cells, and localized to the cell plate during both early and late cytokinesis; furthermore, the TRAPPII complex is involved in the formation of the cell plate in cytokinesis (Smertenko et al., 2017; Garcia et al., 2020). Loss-of-function *tripp* mutants exhibit infertility, dwarfism, and partial photomorphogenesis in the dark, and the *tripp* mutant has reduced polarity of the auxin transporter PIN2, incomplete transverse cell wall formation, and disordered localization of TRAPPII-specific component formation (Garcia et al., 2020; Hughes, 2020). In addition, TRS33 is required for the membrane association of TRS120 and for its localization during cytokinesis (Garcia et al., 2020). The *trs33-1* mutant exhibited shorter roots, stunted growth, and sterility, due to impaired cytokinesis, similar to *trappii* mutants (Thellmann et al., 2010; Garcia et al., 2020). Furthermore, TRAPPII is functionally upstream of several RabA GTPases in *Arabidopsis*, indicating that it can also function as a Rab GEF (Qi et al., 2011; Kalde et al., 2019). These studies indicate that TRAPPII regulates vesicle trafficking and the assembly of cell plates, and it is essential for plant growth and development.

**Table 2:** Characteristics of tethers located in cell plates.

Type	Gene	AGI Gene	Localization	Function	Reference
TRAPPII	TRS120/VAN4	AT5g11040	TGN/EE, CP	Required for cell plate biogenesis during cytokinesis; polar localization of PIN2; mediated exocytosis at the TGN; regulated the post-Golgi trafficking.	Qi et al., 2011; Naramoto et al., 2014; Rybak et al., 2014; Ravikumar et al., 2018; Kalde et al., 2019
	TRS130/ CLUB	AT5g54440	TGN/EE, CP	Required for cell plate biogenesis during cytokinesis; regulating intracellular trafficking; polar localization of PIN2; regulates the post-Golgi trafficking.	Jaber et al., 2010; Qi et al., 2011; Rybak et al., 2014; Ravikumar et al., 2017; Kalde et al., 2019
	TRIPP	AT3g17900	TGN/EE, CP	Involved in cytokinesis; polar localization of PIN2; plant specific component of TRAPPII vesicle transport complex.	Garcia et al., 2020
	TRS33	AT3g05000	TGN/EE, Golgi	Involved in cytokinesis; auxin distribution; polar localization of both PIN1 and PIN2.	Kalde et al., 2019; Garcia et al., 2020; Zhang et al., 2020
Exocyst	SEC3A	AT1g47550	PM, CP, Cytoplasm	As a polarity determinant that links between polarized exocytosis and cell morphogenesis; tethers secretory vesicles to specific domains of the PM.	Zhang et al., 2013; Bloch et al., 2016; Li et al., 2017
	SEC6	AT1g71820	PM, CP, Cytoplasm	Involved in vesicle tethering during cell plate formation; regulate membrane fusion; help to tether the vesicles before fusion; polar auxin transport and PIN protein recycling.	Fendrych et al., 2010; Wu et al., 2013; Tan et al., 2016; Tan et al., 2022
	SEC8	AT3g10380	CP, PM, Cytoplasm	Involved in cytokinesis; involved in recycling of PIN1, PIN2 and the brassinosteroid receptor BRI1 to the PM; involved in the localized deposition of seed coat pectin.	Kulich et al., 2010; Drdová et al., 2013; Janková Drdová et al., 2019
	SEC10	AT5g12370	PM, CP, Cytoplasm	Involved in exocytotic vesicle fusion; PIN protein recycling and polar auxin transport.	Drdová et al., 2013; Zmienko et al., 2020
	SEC15B	At4g02350	PM, CP, Cytoplasm	Involved in cytokinesis; involved in tethering vesicles to the PM.	Rybak et al., 2014; Mayers et al., 2017
	EXO70 A1	AT5g03540	PM, CP, Cytoplasm	Involved in cytokinesis; auxin efflux carrier recycling and polar auxin transport; involved in cell and organ morphogenesis; required for exocyst recruitment to the PM; secretion; EXO70A1 is required for the location of CASP1 at the Casparian Strip Domain (CSD).	Synek et al., 2006; Fendrych et al., 2010; Drdová et al., 2013; Kalmbach et al., 2017; Larson et al., 2020; Synek et al., 2021; Hématy et al., 2022
	EXO84B	At5g49830	PM, CP	Required for Cell plate maturation and cell plate to PM fusion in the final stages of cytokinesis; affects CASP1 localization in CSD and secretion of many integral membrane proteins.	Fendrych et al., 2010 Kulich et al., 2010; Cole et al., 2014; Synek et al., 2021; Hématy et al., 2022

TGN, trans-Golgi network; EE, early endosome; PM, plasma membrane; and CP, cell plate.

Seven exocyst subunits (SEC3A, SEC6, SEC8, SEC10, SEC15, EXO70A1, and EXO84B) are localized to the cell plate (**Table 2**). SEC3A is preferentially expressed in tissues containing dividing and expanding cells (Zhang et al., 2013; Bloch et al., 2016; Li et al., 2017). Moreover, SEC3A-GFP is temporarily located in the early cell plate, disappears during cell plate elongation and reappears in the division wall. In interphase cells, SEC3A-GFP is localized in the cytoplasm and PM, where it forms solid punctate structures (Zhang et al., 2013; Li et al., 2017). At the start of cytokinesis, SEC6-GFP, GFP-SEC8, GFP-SEC15b, and EXO70A1-GFP were tightly associated with the cell plate at the moment of its emergence and were localized to the cell plate as determined by fluorescence; then, during the formation of the cell plate, the signal diminished until it reappeared at the time of cell plate insertion (Fendrych et al., 2010; Rybak et al., 2014; Gu and Rasmussen, 2022). SEC6 localizes to the cell plate, cytoplasm, post-cytokinetic wall, and somewhat to PM as determined by labeling (Fendrych et al., 2010; Tan et al., 2022). Moreover, SEC6 interacts with the SM (Sec1p/Munc18) protein KEULE and may act as a novel molecular link between vesicles and the machinery for membrane fusion; alternatively, it may directly regulate membrane fusion during the formation of plant cell plates (Wu et al., 2013). In addition, pollen-rescued *sec6* mutants (*PRsec6*) form numerous binucleate cells and cell wall stubs in embryonic cells and abnormally dividing guard cells and cell wall stubs in leaf epidermal cells (Wu et al., 2013). Furthermore, the *sec6-1-/+* and *sec6-2-/+* mutants show approximately 15% of pollen grains broken in cytokinesis during pollen mitosis I (PMI) and impaired cell plate formation (Fendrych et al., 2010; Tan et al., 2022). SEC8 localizes to the nascent cell plate and later to the extension region of the cell plate, and it is involved in cytokinesis (Fendrych et al., 2010). *Sec8* mutants show severe dwarfism and male-specific transmission defects, and root cortical cells elongate at a slower rate in shorter elongation zones (Cole et al., 2005; Kulich et al., 2010; Cole et al., 2014). SEC10 is uniformly localized in the PM, cytoplasm, and cell plates; however, the T-DNA insertion mutant of *sec10* shows no obvious phenotypic defects, possibly due to functional redundancy (Fendrych et al., 2010; Drdová et al., 2013; Vukašinović et al., 2014). RFP-SEC15B is primarily localized to the cell plate and punctate structures at or near the PM (Fendrych et al., 2010; Rybak et al., 2014; Mayers et al., 2017). EXO70A1 is involved in cell plate initiation, and the *exo70a1* mutant shows impaired initial cell plate morphology during cell plate assembly (Synek et al., 2006; Fendrych et al., 2010; Synek et al., 2021). EXO84B is required for the maturation of the cell plate, and changes in membrane properties drive the observed changes in polysaccharide composition, because tethering complexes bind distinct populations of vesicles with different cargos to the cell plate or cross wall (Fendrych et al., 2010; Cole et al., 2014). The *exo84b* mutant shows sterile dwarfs, slow growth, infrequent cell divisions, major defects with cell dynamics at the cellular level, leaf-like epidermis with cell wall stubs, highly asymmetric stomata, and incomplete division of stomatal guard cells (Fendrych et al., 2010; Hématy et al., 2022). These results show that the exocyst complex is mainly involved in the initiation and maturation of the cell plate and formation of the new primary cell wall during *Arabidopsis* cytokinesis.

## 4 SNAREs mediate membrane fusion during cell plate formation

SNAREs are responsible for mediating vesicle-to-target membrane fusion, and the *Arabidopsis* genome encodes at least 64 SNAREs (Sanderfoot, 2007; Luo et al., 2022). SNARE proteins are classified as Q-SNARE or R-SNARE according to the core SNARE complex residues (glutamine and arginine, respectively) that contribute to structural assembly. Q-SNAREs are further divided into Qa-, Qb-, Qc-, and Qbc-SNAREs (Fasshauer et al., 1998; Bock et al., 2001; Lipka et al., 2007; Luo et al., 2022). SNAREs are involved in a variety of biological processes such as auxin polar transport, vesicle trafficking, autophagy, gravitropism, and biotic and abiotic stress responses (Saito and Ueda, 2009; Larson et al., 2014; Won and Kim, 2020). In addition, SNARE protein-mediated membrane fusion promotes cell plate formation in dividing cells (Lukowitz et al., 1996; El Kasmi et al., 2013; Park et al., 2018). SNAREs are the core machinery mediating membrane fusion, and an important step in membrane fusion is the formation of *trans*-SNARE complexes, which connect cell membranes so that they can fuse together (Jahn and Scheller, 2006; Saito and Ueda, 2009). Each functional SNARE complex requires two or three Q-SNAREs and one R-SNARE to generate fusion complexes based on homology to synaptic SNAREs; furthermore, cell plate formation requires vesicle fusion mediated by SNAREs and their regulators (Südhof and Rothman, 2009; Luo et al., 2022). Initially, inactive cis-SNARE complexes assemble on the ER and traffic along the secretory pathway through the Golgi and TGN to the cell division plane (Karnahl et al., 2017). In the cell division plane, the *cis*-SNARE complex is broken by NSF ATPase, and Qa-SNARE KNOLLE interacts with the Sec1p/Munc18 (SM) protein KEULE to keep KNOLLE in an open conformation, thereby promoting the formation of *trans*-SNARE complexes on adjacent vesicles by KNOLLE and its SNARE partners (Waizenegger et al., 2000; Assaad et al., 2001; Südhof and Rothman, 2009; Carr and Rizo, 2010; Park et al., 2012). KEULE also interacts with the exocyst and it provides a direct link between tethering and the formation of *trans*-SNARE complexes (Wu et al., 2013). To date, four complete SNARE complexes have been found to mediate membrane fusion during *Arabidopsis* cytokinesis (**Figure 2**) (El Kasmi et al., 2013; Park et al., 2018).

Five Qa-SNAREs (KNOLLE, SYP132, SYP121, SYP122, and SYP31) are involved in vesicle fusion during cell plate formation (**Table 3**). The Qa-SNARE KNOLLE is a cytokinesis-specific Syntaxin that accumulates in the TGN during early mitosis, then localizes to the cell plate during cytokinesis, and degrades *via* multivesicular bodies (MVBs) after completion of the newly formed PM (Lauber et al., 1997; Stierhof and El Kasmi, 2010; Reichardt et al., 2011). KNOLLE is expressed in a cell cycle-dependent manner and it mediates cell plate formation through vesicle fusion in the cell division plane (Lukowitz et al., 1996; Lauber et al., 1997). Moreover, KNOLLE is required for both somatic cytokinesis and endosperm cellularization (Park et al., 2018). *Knolle* mutant embryos develop abnormally and exhibit severe cytokinesis defects, such as incomplete cell walls and two or more nuclei, leading to seedlings lacking functional meristems, forming plaques of necrotic tissue, and eventually dying (Lukowitz et al., 1996). Evolutionarily ancient and originating from the algal ancestor Qa-SNARE, SYP132 localizes to the PM and cell plate. Moreover, SYP132 is essential for secretion and it plays an important role in membrane fusion during cytokinesis. In addition, the *syp132* mutant can have cytokinesis defects, such as multinucleated cells, cell wall stubs, cell wall debris, and nonfused vesicle bands on the cell division plane (Park et al., 2018). The Qa-SNARE SYP121 is localized to the PM and TGN, accumulates strongly on the cell plate during cytokinesis, and constitutively cycles between the PM and endosomes; however, the accumulation of the Qa-SNARE SYP122 in cell plates is relatively weak compared with that of SYP121 (Reichardt et al., 2011; Karnik et al., 2015; Liu et al., 2022). The Qa-SNARE SYP31 localizes to cell plates in *Arabidopsis* suspension cells, indicating that it is involved in cell plate formation during somatic cytokinesis. Furthermore, AtCDC48 interacts specifically with SYP31 in an ATP-dependent manner, but not with KNOLLE, which may be necessary for the fusion of “other” secretory membranes in the cell division plane (Rancour et al., 2002). In addition, Qa-SNAREs SYP31 and SYP32 regulate membrane trafficking and Golgi morphology during pollen development, and *syp31/+ syp32/+* double mutants show developmental defects in pollen with abnormal cell plate formation during PMI (Rancour et al., 2002; Rui et al., 2021).

**Table 3:** Characteristics of SNAREs located in cell plates.

Type	Gene	AGI Gene	Localization	Function	Reference
Qa	KNOLLE /SYP111	AT1g08560	TGN/EE, MVB, CP	Membrane fusion during cell plate formation; secretory vesicles trafficking on PM.	Lauber et al., 1997; Völker et al., 2001; El Kasmi et al., 2013; Park et al., 2018
	SYP132	AT5g08080	PM, CP	Membrane fusion in cytokinesis; involved in secretory pathways; vesicular trafficking at the PM; promotes PM H^+^-ATPase trafficking; response to bacterial pathogens.	Ichikawa et al., 2014; Park et al., 2018; Xia et al., 2019; Baena et al., 2022
	SYP121 /PEN1	AT3g11820	TGN/EE, PM, CP	As a negative regulator in innate immunity; affect K^+^ channel and promote K^+^ absorption; vesicular trafficking at the PM.	Honsbein et al., 2009 Reichardt et al., 2011; Liu et al., 2022; Cui et al., 2022; Rubiato et al., 2022
	SYP122	AT3g52400	PM, CP	Vesicular trafficking at the PM; involved in secretion; negative regulation of programmed cell death.	Zhang et al., 2007; Liu et al., 2022; Rubiato et al., 2022
	SYP31	At5g05760	Golgi, CP	Involved in cell division and secretion pathways; Regulation of Golgi morphology; involved in ER-Golgi trafficking.	Rancour et al., 2002; Bubeck et al., 2008; Rui et al., 2021
Qb	NPSN11	AT2g35190	CP, PM, E	Membrane fusion during cell division; involved in cell secretion.	Zheng et al., 2002; El Kasmi et al., 2013; Park et al., 2018
Qc	SYP71	At3g09740	CP, PM, E, ER	Membrane fusion during cell plate formation; involved in vesicular trafficking to ER.	Suwastika et al., 2008; El Kasmi et al., 2013; Park et al., 2018
Qbc	SNAP33	AT5g61210	Cytoplasm, E, PM, CP	Membrane fusion during cell plate formation; innate immune and abiotic stress responses; involved in the secretion process.	Heese et al., 2001; El Kasmi et al., 2013; Park et al., 2018; Won and Kim, 2020
R	VAMP721	AT1g04750	TGN/EE, E, PM, CP	Involved in cell plate formation; regulating auxin transport and auxin distribution; regulation of K^+^ uptake; involved in exocytosis and response to ER stress; Regulates the cell secretory pathway.	Yun et al., 2013; Zhang et al., 2011; Zhang et al., 2020; Zhang et al., 2021; Kim et al., 2019; Larson et al., 2020
	VAMP722	AT2g33120	TGN/EE, E, PM, CP		
	SEC22	At1g11890	Nuclear envelope, ER, CP	Cytoskeleton organization and stability; works in the early secretory pathway; ER-Golgi trafficking.	Chatre et al., 2005; El-Kasmi et al., 2011; Guan et al., 2021

E, endosome; TGN, trans-Golgi network; EE, early endosome; ER, endoplasmic reticulum; MVBs, multivesicular bodies; PM, plasma membrane; and CP, cell plate.

The other three SNAREs, Qb-SNARE (NPSN11), Qc-SNARE (SYP71), and Qbc-SNARE (SNAP33), are involved in vesicle fusion during cell plate formation (**Table 3**). NPSN11 is a novel plant-specific Qb-SNARE that is highly expressed in tissues with active cell division and localized to the cell plate during cytokinesis. SYP71 of Qc-SNARE located at the PM, endosome, endoplasmic reticulum, and cell plate (El Kasmi et al., 2013). SNAP33 of Qbc-SNARE is a widely expressed membrane-associated protein localized to the PM, endosome, and cell plates (Heese et al., 2001; El Kasmi et al., 2013). Furthermore, SNAP33 is involved in membrane fusion during cell plate formation and plays a role in cytokinesis. In addition, *snap33* mutant seedlings show minor cytokinesis defects, and only later cotyledon lesions lead to lethality (Heese et al., 2001). S*nap33 npsn11* double mutant embryos show severe defects in cytokinesis and impaired cell plate formation, and the *snap33 syp71* double mutant has severe cytokinesis-related phenotypes (El Kasmi et al., 2013).

Three R-SNAREs (VAMP721, VAMP722, and SEC22) are involved in vesicle fusion during cell plate formation (**Table 3**). VAMP721 and VAMP722 are localized to the PM, the TGN/EE, and preferentially to expanding cell plates during cytokinesis. Moreover, VAMP721 and VAMP722 mediate PM secretion and vesicle fusion on the cell plate (Zhang et al., 2011). Furthermore, the *vamp721 vamp722* mutant shows cell wall stub and delayed expansion of the cell plate, and the seedlings of the double mutant are underdeveloped and eventually show lethal dwarfing phenotypes (Zhang et al., 2011; Zhang et al., 2021). The third R-SNARE, SEC22 is visible at the plane of cell division during cytokinesis, where it colocalizes with KNOLLE and works in the early secretory pathway, which is essential for the integrity of the ER network and the Golgi complex (Chatre et al., 2005; El-Kasmi et al., 2011; Guan et al., 2021). In addition, *sec22-4* mutants have delayed germination, short primary roots, dwarfing and partial abortion, and changes in the shape of their trichomes, pavement cells, and stomatal morphology (El-Kasmi et al., 2011). Taken together, these results show that multiple types of SNAREs are required to mediate the fusion of vesicles during cell plate formation.

## 5 Perspective

Endomembrane trafficking undergoes various transitions during cell plate formation. During the early stages of cell plate formation, late secretory vesicles derived from the TGN migrate along the phragmoplast toward the cell equator. Dynamic reorganization of the cytoplasm drives lateral expansion of the cell plate, resulting in the shedding of vesicles at the edges of the growing cell plate. Then, the constant flow of vesicles toward the edge of the newly formed cell plate causes the cell plate to swell and eventually fuse with the original PM (Mayer and Jürgens, 2004). During cell plate formation, Rab GTPases, tethers, and SNAREs are localized to certain membranes and function in particular vesicle trafficking events; furthermore, they are crucial regulators of membrane targeting, identity, and fusion (Chow et al., 2008; Martinière and Moreau, 2020; Risselada and Mayer, 2020). Rab GTPases are master regulators of membrane trafficking, regulating the transport of vesicles during cell plate formation (Davis et al., 2016; Minamino and Ueda, 2019). The two tethering complexes (TRAPPII and exocyst) physically interact to coordinate the formation of cell plates during cytokinesis. The TRAPPII complex marks the cell plate throughout cytokinesis and is required for cell plate biogenesis; however, the exocyst is required for maturation of the cell plate (Rybak et al., 2014; Boruc and Van Damme, 2015). Tethering proteins provide specificity for targeting, and vesicle tethering initiates SNARE-dependent fusion of membrane vesicles to form cell plates (Yu and Hughson, 2010). Rab GTPases, tethers, and SNAREs function synergistically to promote vesicle fusion, which increases the specificity and efficiency of membrane fusion (Ebine et al., 2008; Ohya et al., 2009; Boutté et al., 2010; Ebine et al., 2011).

Rab GTPases and SNARE complexes are functionally linked by tethering complexes, which mediate the tethering of these two membranes components prior to membrane fusion (Wickner and Schekman, 2008; Takemoto et al., 2018). RabF1 localizes to the PM, where it plays a regulatory role in the formation of a SNARE complex containing endosome associated VAMP727 and PM-localized SYP121 (Ebine et al., 2011). In addition, RabA, B, D, and E GTPases are identified in the TRAPPII interactome, and TRAPPII functions as the upstream of RabA2a, which is likely to behave as a GEF for the RabA2a GTPase (Kalde et al., 2019). Furthermore, tethers mediating the physical contact between vesicles and target membranes, together with Rab GTPases, play a key role in determining the specificity of vesicle targeting and fusion events (Cai et al., 2007). Therefore, Rab GTPases, tethers, and SNAREs may coordinate the regulation of the cell plate formation (**Tables 1**, **2**, **3**; **Figure 3A**). However, the spatialization of the three on the cell plate is not completely clear, future research should focus on determining their precise spatiotemporal location on the cell plate and the interaction network between them. Besides, cell plate formation stage IV peripheral microtubules come in contact with the cell cortex and then cell plate extends fusion tubes connecting to the cell plate fusion site at the PM, and fuses with the PM (Boruc and Van Damme, 2015; Smertenko et al., 2017; Rodriguez-Furlan et al., 2019). Thus, to reveal the fine coordination between Rab GTPases, tethers, and SNAREs, the mechanism required for fusion of the cell plate with the PM remains to be identified, and how proteins diffused from the cell plate are recovered or degraded at this stage remains to be studied (**Figure 1**).

The process of cell plate localization and formation is highly complicated, and some molecular mechanisms are well understood, but many questions remain to be answered. How are vesicles transported along the phragmoplast to the cell division plane for delicate tethering and fusion? Many Rab GTPases, tethers, and SNAREs members are located to the cell plate, and how do they each coordinate the regulation of cell plate formation? Is there a difference in the molecular composition and cargo of the vesicles that are involved in the assembly of cell plates? It is not clear whether vesicles carrying different cargoes destined for the cell plate are regulated by different Rab GTPases. The identification of more proteins and mechanisms involved in cell plate formation remains a goal. A better understanding of the molecular mechanism of cell plate formation will be gained over time with further research.

## Author contributions

YS, CL, YX, and DQ wrote the manuscript. All authors contributed to the article and approved the submitted version.

## References

[B1] Ahn C. S. Han J. A. Pai H. S. (2013). Characterization of *in vivo* functions of *Nicotiana benthamiana* RabE1. Planta 237, 161–172. doi: 10.1007/s00425-012-1760-5 23001196

[B2] Ambastha V. Matityahu I. Tidhar D. Leshem Y. (2021). RabA2b overexpression alters the plasma-membrane proteome and improves drought tolerance in *Arabidopsis* . Front. Plant Sci. 12, 738694. doi: 10.3389/fpls.2021.738694 34691115 PMC8526897

[B3] Asaoka R. Uemura T. Ito J. Fujimoto M. Ito E. Ueda T. . (2013). *Arabidopsis* RABA1 GTPases are involved in transport between the *trans*-golgi network and the plasma membrane, and are required for salinity stress tolerance. Plant J. 73, 240–249. doi: 10.1111/tpj.12023 22974509

[B4] Assaad F. F. Huet Y. Mayer U. Jurgens G. (2001). The cytokinesis gene KEULE encodes a Sec1 protein that binds the syntaxin KNOLLE. J. Cell Biol. 152, 531–543. doi: 10.1083/jcb.152.3.531 11157980 PMC2195996

[B5] Baena G. Xia L. Waghmare S. Karnik R. (2022). SNARE SYP132 mediates divergent traffic of plasma membrane h^+^-ATPase AHA1 and antimicrobial PR1 during bacterial pathogenesis. Plant Physiol. 189, 1639–1661. doi: 10.1093/plphys/kiac149 35348763 PMC9237740

[B6] Baluska F. Liners F. Hlavacka A. Schlicht M. Van Cutsem P. McCurdy D. W. . (2005). Cell wall pectins and xyloglucans are internalized into dividing root cells and accumulate within cell plates during cytokinesis. Protoplasma 225, 141–155. doi: 10.1007/s00709-005-0095-5 16228896

[B7] Berson T. von Wangenheim D. Takáč T. Šamajová O. Rosero A. Ovečka M. . (2014). *Trans*-golgi network localized small GTPase RabA1d is involved in cell plate formation and oscillatory root hair growth. BMC Plant Biol. 14, 252. doi: 10.1186/s12870-014-0252-0 25260869 PMC4180857

[B8] Bloch D. Pleskot R. Pejchar P. Potocky M. Trpkosova P. Cwiklik L. . (2016). Exocyst SEC3 and phosphoinositides define sites of exocytosis in pollen tube initiation and growth. Plant Physiol. 172, 980–1002. doi: 10.1104/pp.16.00690 27516531 PMC5047084

[B9] Bock J. B. Matern H. T. Peden A. A. Scheller R. H. (2001). A genomic perspective on membrane compartment organization. Nature 409, 839–841. doi: 10.1038/35057024 11237004

[B10] Boruc J. Van Damme D. (2015). Endomembrane trafficking overarching cell plate formation. Curr. Opin. Plant Biol. 28, 92–98. doi: 10.1094/MPMI.2001.14.6.695 26485667

[B11] Bottanelli F. Foresti O. Hanton S. Denecke J. (2011). Vacuolar transport in *tobacco* leaf epidermis cells involves a single route for soluble cargo and multiple routes for membrane cargo. Plant Cell 23, 3007–3025. doi: 10.1105/tpc.111.085480 21856792 PMC3180807

[B12] Boutté Y. Frescatada-Rosa M. Men S. Chow C. M. Ebine K. Gustavsson A. . (2010). Endocytosis restricts *Arabidopsis* KNOLLE syntaxin to the cell division plane during late cytokinesis. EMBO J. 29, 546–558. doi: 10.1038/emboj.2009.363 19959995 PMC2789941

[B13] Bubeck J. Scheuring D. Hummel E. Langhans M. Viotti C. Foresti O. . (2008). The syntaxins SYP31 and SYP81 control ER-golgi trafficking in the plant secretory pathway. Traffic 9, 1629–1652. doi: 10.1111/j.1600-0854.2008.00803.x 18764818

[B14] Cai H. Reinisch K. Ferro-Novick S. (2007). Coats, tethers, rabs, and SNAREs work together to mediate the intracellular destination of a transport vesicle. Dev. Cell 12, 671–682. doi: 10.1016/j.devcel.2007.04.005 17488620

[B15] Carr C. M. Rizo J. (2010). At The junction of SNARE and SM protein function. Curr. Opin. Plant Biol. 22, 488–495. doi: 10.1016/j.ceb.2010.04.006 PMC2923694 20471239

[B16] Chatre L. Brandizzi F. Hocquellet A. Hawes C. Moreau P. (2005). Sec22 and Memb11 are v-SNAREs of the anterograde endoplasmic reticulum-golgi pathway in *tobacco* leaf epidermal cells. Plant Physiol. 139, 1244–1254. doi: 10.1104/pp.105.067447 16244155 PMC1283762

[B17] Chow C. M. Neto H. Foucart C. Moore I. (2008). Rab-A2 and rab-A3 GTPases define a *trans*-golgi endosomal membrane domain in *Arabidopsis* that contributes substantially to the cell plate. Plant Cell 20, 101–123. doi: 10.1105/tpc.107.052001 18239134 PMC2254926

[B18] Cole R. A. McInally S. A. Fowler J. E. (2014). Developmentally distinct activities of the exocyst enable rapid cell elongation and determine meristem size during primary root growth in *Arabidopsis* . BMC Plant Biol. 14, 386. doi: 10.1186/s12870-014-0386-0 25551204 PMC4302519

[B19] Cole R. A. Synek L. Zarsky V. Fowler J. E. (2005). SEC8, a subunit of the putative *Arabidopsis* exocyst complex, facilitates pollen germination and competitive pollen tube growth. Plant Physiol. 138, 2005–2018. doi: 10.1104/pp.105.062273 16040664 PMC1183391

[B20] Cui S. Fukao Y. Mano S. Yamada K. Hayashi M. Nishimura M. (2013). Proteomic analysis reveals that the rab GTPase RabE1c is involved in the degradation of the peroxisomal protein receptor PEX7 (peroxin 7). J. Biol. Chem. 288, 6014–6023. doi: 10.1074/jbc.M112.438143 23297417 PMC3581416

[B21] Cui X. Wang S. Huang Y. Ding X. Wang Z. Zheng L. . (2022). *Arabidopsis* SYP121 acts as an ROP2 effector in the regulation of root hair tip growth. Mol. Plant 15, 1008–1023. doi: 10.1016/j.molp.2022.04.008 35488430

[B22] Cui Y. Zhao Q. Gao C. Ding Y. Zeng Y. Ueda T. . (2014). Activation of the Rab7 GTPase by the MON1-CCZ1 complex is essential for PVC-to-vacuole trafficking and plant growth in *Arabidopsis* . Plant Cell 26, 2080–2097. doi: 10.1105/tpc.114.123141 24824487 PMC4079370

[B23] Davis D. J. McDowell S. C. Park E. Hicks G. Wilkop T. E. Drakakaki G. (2016). The RAB GTPase RABA1e localizes to the cell plate and shows distinct subcellular behavior from RABA2a under endosidin 7 treatment. Plant Signal. Behav. 11, e984520. doi: 10.4161/15592324.2014.984520 27408949 PMC4883879

[B24] Dhonukshe P. Baluska F. Schlicht M. Hlavacka A. Samaj J. Friml J. . (2006). Endocytosis of cell surface material mediates cell plate formation during plant cytokinesis. Dev. Cell 10, 137–150. doi: 10.1016/j.devcel.2005 16399085

[B25] Drdová E. J. Synek L. Pečenková T. Hála M. Kulich I. Fowler J. E. . (2013). The exocyst complex contributes to PIN auxin efflux carrier recycling and polar auxin transport in *Arabidopsis* . Plant J. 73, 709–719. doi: 10.1111/tpj.12074 23163883

[B26] Ebine K. Fujimoto M. Okatani Y. Nishiyama T. Goh T. Ito E. . (2011). A membrane trafficking pathway regulated by the plant-specific RAB GTPase ARA6. Nat. Cell Biol. 13, 853–859. doi: 10.1038/ncb2270 21666683

[B27] Ebine K. Miyakawa N. Fujimoto M. Uemura T. Nakano A. Ueda T. (2012). Endosomal trafficking pathway regulated by ARA6, a RAB5 GTPase unique to plants. Small GTPases 3, 23–27. doi: 10.4161/sgtp.18299 22710734 PMC3398913

[B28] Ebine K. Okatani Y. Uemura T. Goh T. Shoda K. Niihama M. . (2008). A SNARE complex unique to seed plants is required for protein storage vacuole biogenesis and seed development of *Arabidopsis thaliana* . Plant Cell 20, 3006–3021. doi: 10.1105/tpc.107.057711 18984676 PMC2613668

[B29] El Kasmi F. Krause C. Hiller U. Stierhof Y. D. Mayer U. Conner L. . (2013). SNARE complexes of different composition jointly mediate membrane fusion in *Arabidopsis* cytokinesis. Mol. Biol. Cell 24, 1593–1601. doi: 10.1091/mbc.E13-02-0074 23515225 PMC3655819

[B30] El-Kasmi F. Pacher T. Strompen G. Stierhof Y. D. Muller L. M. Koncz C. . (2011). *Arabidopsis* SNARE protein SEC22 is essential for gametophyte development and maintenance of golgi-stack integrity. Plant J. 66, 268–279. doi: 10.1111/j.1365-313X.2011.04487.x 21205036

[B31] Elliott L. Moore I. Kirchhelle C. (2020). Spatio-temporal control of post-golgi exocytic trafficking in plants. J. Cell Sci. 133, jcs237065. doi: 10.1242/jcs.237065 32102937

[B32] Euteneuer U. McIntosh J. R. (1980). Polarity of midbody and phragmoplast microtubules. J. Cell Biol. 87, 509–515. doi: 10.1083/jcb.87.2.509 7430255 PMC2110749

[B33] Fasshauer D. Sutton R. B. Brunger A. T. Jahn R. (1998). Conserved structural features of the synaptic fusion complex: SNARE proteins reclassified as q-and r-SNAREs. Proc. Natl. Acad. U. S. A. 95, 15781–15786. doi: 10.1073/pnas.95.26.15781 PMC28121 9861047

[B34] Fendrych M. Synek L. Pecenkova T. Toupalova H. Cole R. Drdova E. . (2010). The *Arabidopsis* exocyst complex is involved in cytokinesis and cell plate maturation. Plant Cell 22, 3053–3065. doi: 10.1105/tpc.110.074351 20870962 PMC2965533

[B35] Feraru E. Feraru M. I. Asaoka R. Paciorek T. De Rycke R. Tanaka H. . (2012). BEX5/RabA1b regulates *trans*-golgi network-to-plasma membrane protein trafficking in *Arabidopsis* . Plant Cell 24, 3074–3086. doi: 10.1105/tpc.112.098152 22773752 PMC3426133

[B36] Garcia V. J. Xu S. L. Ravikumar R. Wang W. Elliott L. Gonzalez E. . (2020). TRIPP is a plant-specific component of the *Arabidopsis* TRAPPII membrane trafficking complex with important roles in plant development. Plant Cell 32, 2424–2443. doi: 10.1105/tpc.20.00044 32371545 PMC7346556

[B37] Geldner N. Denervaud-Tendon V. Hyman D. L. Mayer U. Stierhof Y. D. Chory J. (2009). Rapid, combinatorial analysis of membrane compartments in intact plants with a multicolor marker set. Plant J. 59, 169–178. doi: 10.1111/j.1365-313X.2009.03851.x 19309456 PMC4854200

[B38] Gu Y. Rasmussen C. G. (2022). Cell biology of primary cell wall synthesis in plants. Plant Cell 34, 103–128. doi: 10.1093/plcell/koab249 34613413 PMC8774047

[B39] Guan L. Yang S. Li S. Liu Y. Liu Y. Yang Y. . (2021). AtSEC22 regulates cell morphogenesis *via* affecting cytoskeleton organization and stabilities. Front. Plant Sci. 12, 635732. doi: 10.3389/fpls.2021.635732 34149743 PMC8211912

[B40] He B. Guo W. (2009). The exocyst complex in polarized exocytosis. Curr. Opin. Cell Biol. 21, 537–542. doi: 10.1016/j.ceb.2009.04.007 19473826 PMC2725219

[B41] He M. Lan M. Zhang B. Zhou Y. Wang Y. Zhu L. . (2018). Rab-H1b is essential for trafficking of cellulose synthase and for hypocotyl growth in *Arabidopsis thaliana* . J. Integr. Plant Biol. 60, 1051–1069. doi: 10.1111/jipb.12694 29975455

[B42] Heese M. Gansel X. Sticher L. Wick P. Grebe M. Granier F. . (2001). Functional characterization of the KNOLLE-interacting t-SNARE AtSNAP33 and its role in plant cytokinesis. J. Cell Biol. 155, 239–249. doi: 10.1083/jcb.200107126 11591731 PMC2198836

[B43] Heider M. R. Munson M. (2012). Exorcising the exocyst complex. Traffic 13, 898–907. doi: 10.1111/j.1600-0854.2012.01353.x 22420621 PMC3374049

[B44] Hématy K. De Bellis D. Wang X. Mähönen A. P. Geldner N. (2022). Analysis of exocyst function in endodermis reveals its widespread contribution and specificity of action. Plant Physiol. 189, 557–566. doi: 10.1093/plphys/kiac019 35099565 PMC9157074

[B45] Hong W. Lev S. (2014). Tethering the assembly of SNARE complexes. Trends Cell Biol. 24, 35–43. doi: 10.1016/j.tcb.2013.09.006 24119662

[B46] Honsbein A. Sokolovski S. Grefen C. Campanoni P. Pratelli R. Paneque M. . (2009). A tripartite SNARE-k^+^ channel complex mediates in channel-dependent k^+^ nutrition in *Arabidopsis* . Plant Cell 21, 2859–2877. doi: 10.1105/tpc.109.066118 19794113 PMC2768940

[B47] Hughes P. W. (2020). It's a TRAPP! *Arabidopsis* transport protein particle (TRAPP) complexes contain a novel plant-specific subunit. Plant Cell 32, 2081–2082. doi: 10.1105/tpc.20.00375 32409320 PMC7346575

[B48] Hutagalung A. H. Novick P. J. (2011). Role of rab GTPases in membrane traffic and cell physiology. Physiol. Rev. 91, 119–149. doi: 10.1152/physrev.00059.2009 21248164 PMC3710122

[B49] Ichikawa M. Hirano T. Enami K. Fuselier T. Kato N. Kwon C. . (2014). Syntaxin of plant proteins SYP123 and SYP132 mediate root hair tip growth in *Arabidopsis thaliana* . Plant Cell Physiol. 55, 790–800. doi: 10.1093/pcp/pcu048 24642714

[B50] Inada N. Ebine K. Ito E. Nakano A. Ueda T. (2017). Constitutive activation of plant-specific RAB5 GTPase confers increased resistance against adapted powdery mildew fungus. Plant Biotechnol. (Tokyo) 34, 89–95. doi: 10.5511/plantbotechnology.17.0501a 31275013 PMC6543761

[B51] Ito E. Uemura T. Ueda T. Nakano A. (2016). Distribution of RAB5-positive multivesicular endosomes and the *trans*-golgi network in root meristematic cells of *Arabidopsis thaliana* . Plant Biotechnol. (Tokyo) 33, 281–286. doi: 10.5511/plantbiotechnology.16.0218a 31367184 PMC6637257

[B52] Jaber E. Thiele K. Kindzierski V. Loderer C. Rybak K. Jurgens G. . (2010). A putative TRAPPII tethering factor is required for cell plate assembly during cytokinesis in *Arabidopsis* . New Phytol. 187, 751–763. doi: 10.1111/j.1469-8137.2010.03331.x 20609115

[B53] Jahn R. Lang T. Südhof T. C. (2003). Membrane fusion. Cell 112, 519–533. doi: 10.1016/s0092-8674(03)00112-0 12600315

[B54] Jahn R. Scheller R. H. (2006). SNAREs-engines for membrane fusion. Nat. Rev. Mol. Cell Biol. 7, 631–643. doi: 10.1038/nrm2002 16912714

[B55] Janková Drdová E. Klejchová M. Janko K. Hála M. Soukupová H. Cvrčková F. . (2019). Developmental plasticity of *Arabidopsis* hypocotyl is dependent on exocyst complex function. J. Exp. Bot. 70, 1255–1265. doi: 10.1093/jxb/erz005 30649396 PMC6382343

[B56] Jia T. Gao C. Cui Y. Wang J. Ding Y. Cai Y. . (2013). ARA7(Q69L) expression in transgenic *Arabidopsis* cells induces the formation of enlarged multivesicular bodies. J. Exp. Bot. 64, 2817–2829. doi: 10.1093/jxb/ert125 23682115 PMC3697957

[B57] Jia P. F. Xue Y. Li H. J. Yang W. C. (2018). Golgi-localized LOT regulates *trans*-golgi network biogenesis and pollen tube growth. Proc. Natl. Acad. Sci. U. S. A. 115, 12307–12312. doi: 10.1073/pnas.1809206115 30413616 PMC6275481

[B58] Johansen J. N. Chow C. M. Moore I. Hawes C. (2009). AtRAB-H1b and AtRAB-H1c GTPases, homologues of the yeast Ypt6, target reporter proteins to the golgi when expressed in nicotiana tabacum and *Arabidopsis thaliana* . J. Exp. Bot. 60, 3179–3193. doi: 10.1093/jxb/erp153 19454595

[B59] Jürgens G. (2005). Cytokinesis in higher plants. Annu. Rev. Plant Biol. 56, 281–299. doi: 10.1146/annurev.arplant.55.031903.141636 15862097

[B60] Kalde M. Elliott L. Ravikumar R. Rybak K. Altmann M. Klaeger S. . (2019). Interactions between transport protein particle (TRAPP) complexes and rab GTPases in *Arabidopsis* . Plant J. 100, 279–297. doi: 10.1111/tpj.14442 31264742

[B61] Kalmbach L. Hematy K. De Bellis D. Barberon M. Fujita S. Ursache R. . (2017). Transient cell-specific EXO70A1 activity in the CASP domain and casparian strip localization. Nat. Plants 3, 17058. doi: 10.1038/nplants.2017.58 28436943

[B62] Karnahl M. Park M. Mayer U. Hiller U. Jurgens G. (2017). ER assembly of SNARE complexes mediating formation of partitioning membrane in *Arabidopsis* cytokinesis. Elife 6, e25327. doi: 10.7554/eLife.25327 28525316 PMC5438246

[B63] Karnik R. Zhang B. Waghmare S. Aderhold C. Grefen C. Blatt M. R. (2015). Binding of SEC11 indicates its role in SNARE recycling after vesicle fusion and identifies two pathways for vesicular traffic to the plasma membrane. Plant Cell 27, 675–694. doi: 10.1105/tpc.114.134429 25747882 PMC4558655

[B64] Kim S. Choi Y. Kwon C. Yun H. S. (2019). Endoplasmic reticulum stress-induced accumulation of VAMP721/722 requires CALRETICULIN 1 and CALRETICULIN 2 in *Arabidopsis* . J. Integr. Plant Biol. 61, 974–980. doi: 10.1111/jipb.12728 30280512

[B65] Kirchhelle C. Chow C. M. Foucart C. Neto H. Stierhof Y. D. Kalde M. . (2016). The specification of geometric edges by a plant rab GTPase is an essential cell-patterning principle during organogenesis in *Arabidopsis* . Dev. Cell 36, 386–400. doi: 10.1016/j.devcel.2016.01.020 26906735 PMC4766369

[B66] Kirchhelle C. Garcia-Gonzalez D. Irani N. G. Jerusalem A. Moore I. (2019). Two mechanisms regulate directional cell growth in *Arabidopsis* lateral roots. Elife 8, e47988. doi: 10.7554/eLife.47988 31355749 PMC6748828

[B67] Kotzer A. M. Brandizzi F. Neumann U. Paris N. Moore I. Hawes C. (2004). AtRabF2b (Ara7) acts on the vacuolar trafficking pathway in *tobacco* leaf epidermal cells. J. Cell Sci. 117, 6377–6389. doi: 10.1242/jcs.01564 15561767

[B68] Koumandou V. L. Dacks J. B. Coulson R. M. Field M. C. (2007). Control systems for membrane fusion in the ancestral eukaryote; evolution of tethering complexes and SM proteins. BMC Evol. Biol. 7, 29. doi: 10.1186/1471-2148-7-29 17319956 PMC1810245

[B69] Kulich I. Cole R. Drdova E. Cvrckova F. Soukup A. Fowler J. . (2010). *Arabidopsis* exocyst subunits SEC8 and EXO70A1 and exocyst interactor ROH1 are involved in the localized deposition of seed coat pectin. New Phytol. 188, 615–625. doi: 10.1111/j.1469-8137.2010.03372.x 20618910

[B70] Larson E. R. Domozych D. S. Tierney M. L. (2014). SNARE VTI13 plays a unique role in endosomal trafficking pathways associated with the vacuole and is essential for cell wall organization and root hair growth in. Arabidopsis. Ann. Bot. 114, 1147–1159. doi: 10.1093/aob/mcu041 24737717 PMC4195547

[B71] Larson E. R. Ortmannová J. Donald N. A. Alvim J. Blatt M. R. Žárský V. (2020). Synergy among exocyst and SNARE interactions identifies a functional hierarchy in secretion during vegetative growth. Plant Cell 32, 2951–2963. doi: 10.1105/tpc.20.00280 32699172 PMC7474273

[B72] Lauber M. H. Waizenegger I. Steinmann T. Schwarz H. Mayer U. Hwang I. . (1997). The *Arabidopsis* KNOLLE protein is a cytokinesis-specific syntaxin. J. Cell Biol. 139, 1485–1493. doi: 10.1083/jcb.139.6.1485 9396754 PMC2132613

[B73] Lee Y. R. Giang H. M. Liu B. (2001). A novel plant kinesin-related protein specifically associates with the phragmoplast organelles. Plant Cell 13, 2427–2439. doi: 10.1105/tpc.010225 11701879 PMC139462

[B74] Lee G. J. Sohn E. J. Lee M. H. Hwang I. (2004). The *Arabidopsis* rab5 homologs rha1 and ara7 localize to the prevacuolar compartment. Plant Cell Physiol. 45, 1211–1220. doi: 10.1093/pcp/pch142 15509844

[B75] Li Y. Tan X. Wang M. Li B. Zhao Y. Wu C. . (2017). Exocyst subunit SEC3A marks the germination site and is essential for pollen germination in *Arabidopsis thaliana* . Sci. Rep. 7, 40279. doi: 10.1038/srep40279 28074928 PMC5225640

[B76] Lipka V. Kwon C. Panstruga R. (2007). SNARE-ware: the role of SNARE-domain proteins in plant biology. Annu. Rev. Cell Dev. Biol. 23, 147–174. doi: 10.1146/annurev.cellbio.23.090506.123529 17506694

[B77] Liu M. Rubiato H. M. Nielsen M. E. (2022). Mobility of the syntaxin PEN1 in *Arabidopsis* reflects functional specialization of the conserved SYP12 clade. Plant Signal. Behav. 17, 2084–2278. doi: 10.1080/15592324.2022.2084278 PMC9196765 35695087

[B78] Lukowitz W. Mayer U. Jürgens G. (1996). Cytokinesis in the *Arabidopsis* embryo involves the syntaxin-related KNOLLE gene product. Cell 84, 61–71. doi: 10.1016/s0092-8674(00)80993-9 8548827

[B79] Luo C. Shi Y. Xiang Y. (2022). SNAREs regulate vesicle trafficking during root growth and development. Front. Plant Sci. 13, 853251. doi: 10.3389/fpls.2022.85325 35360325 PMC8964185

[B80] Martinière A. Moreau P. (2020). Complex roles of rabs and SNAREs in the secretory pathway and plant development: a never-ending story. J. Microsc. 280, 140–157. doi: 10.1111/jmi.12952 32761815

[B81] Mayer U. Jürgens G. (2004). Cytokinesis: lines of division taking shape. Curr. Opin. Plant Biol. 7, 599–604. doi: 10.1016/j.pbi.2004.07.008 15337104

[B82] Mayers J. R. Hu T. Wang C. Cardenas J. J. Tan Y. Pan J. . (2017). SCD1 and SCD2 form a complex that functions with the exocyst and RabE1 in exocytosis and cytokinesis. Plant Cell 29, 2610–2625. doi: 10.1105/tpc.17.00409 28970336 PMC5774579

[B83] Minamino N. Ueda T. (2019). RAB GTPases and their effectors in plant endosomal transport. Curr. Opin. Plant Biol. 52, 61–68. doi: 10.1016/j.pbi.2019.07.007 31454706

[B84] Müller S. (2019). Plant cell division - defining and finding the sweet spot for cell plate insertion. Curr. Opin. Cell Biol. 60, 9–18. doi: 10.1016/j.ceb.2019.03.006 30999231

[B85] Müller S. Jürgens G. (2016). Plant cytokinesis-no ring, no constriction but centrifugal construction of the partitioning membrane. Semin. Cell Dev. Biol. 53, 10–18. doi: 10.1016/j.semcdb.2015.10.037 26529278

[B86] Naramoto S. Nodzyłski T. Dainobu T. Takatsuka H. Okada T. Friml J. . (2014). VAN4 encodes a putative TRS120 that is required for normal cell growth and vein development in *Arabidopsis* . Plant Cell Physiol. 55, 750–763. doi: 10.1093/pcp/pcu012 24443495

[B87] Nishihama R. Machida Y. (2001). Expansion of the phragmoplast during plant cytokinesis: a MAPK pathway may MAP it out. Curr. Opin. Plant Biol. 4, 507–512. doi: 10.1016/s1369-5266(00)00208-9 11641066

[B88] Ohya T. Miaczynska M. Coskun U. Lommer B. Runge A. Drechsel D. . (2009). Reconstitution of rab-and SNARE-dependent membrane fusion by synthetic endosomes. Nature 459, 1091–1097. doi: 10.1038/nature08107 19458617

[B89] Pang L. Ma Z. Zhang X. Huang Y. Li R. Miao Y. . (2022). The small GTPase RABA2a recruits SNARE proteins to regulate the secretory pathway in parallel with the exocyst complex in *Arabidopsis* . Mol. Plant 15, 398–418. doi: 10.1016/j.molp.2021.11.008 34798312

[B90] Park E. Diaz-Moreno S. M. Davis D. J. Wilkop T. E. Bulone V. Drakakaki G. (2014). Endosidin 7 specifically arrests late cytokinesis and inhibits callose biosynthesis, revealing distinct trafficking events during cell plate maturation. Plant Physiol. 165, 1019–1034. doi: 10.1104/pp.114.241497 24858949 PMC4081319

[B91] Park M. Krause C. Karnahl M. Reichardt I. El Kasmi F. Mayer U. . (2018). Concerted action of evolutionarily ancient and novel SNARE complexes in flowering-plant cytokinesis. Dev. Cell 44, 500–511.e504. doi: 10.1016/j.devcel.2017.12.027 29396117

[B92] Park M. Touihri S. Muller I. Mayer U. Jurgens G. (2012). Sec1/Munc18 protein stabilizes fusion-competent syntaxin for membrane fusion in *Arabidopsis* cytokinesis. Dev. Cell 22, 989–1000. doi: 10.1016/j.devcel.2012.03.002 22595672

[B93] Pereira-Leal J. B. Seabra M. C. (2001). Evolution of the rab family of small GTP-binding proteins. J. Mol. Biol. 313, 889–901. doi: 10.1006/jmbi.2001.5072 11697911

[B94] Prekeris R. (2003). Rabs, rips, FIPs, and endocytic membrane traffic. ScientificWorldJournal. 3, 870–880. doi: 10.1100/tsw.2003.69 14532427 PMC5974852

[B95] Qi X. Kaneda M. Chen J. Geitmann A. Zheng H. (2011). A specific role for *Arabidopsis* TRAPPII in post-golgi trafficking that is crucial for cytokinesis and cell polarity. Plant J. 68, 234–248. doi: 10.1111/j.1365-313X.2011.04681.x 21689172

[B96] Qi X. Zheng H. (2013). Rab-A1c GTPase defines a population of the *trans*-golgi network that is sensitive to endosidin1 during cytokinesis in *Arabidopsis* . Mol. Plant 6, 847–859. doi: 10.1093/mp/sss116 23075992

[B97] Rahni R. Birnbaum K. D. (2016). Plant cell shape: trafficking gets edgy. Dev. Cell 36, 353–354. doi: 10.1016/j.devcel.2016.02.005 26906728

[B98] Rancour D. M. Dickey C. E. Park S. Bednarek S. Y. (2002). Characterization of AtCDC48. evidence for multiple membrane fusion mechanisms at the plane of cell division in plants. Plant Physiol. 130, 1241–1253. doi: 10.1104/pp.011742 12427991 PMC166645

[B99] Ravikumar R. Kalbfuss N. Gendre D. Steiner A. Altmann M. Altmann S. . (2018). Independent yet overlapping pathways ensure the robustness and responsiveness of *trans*-golgi network functions in *Arabidopsis* . Development 145, dev169201. doi: 10.1242/dev.169201 30404777

[B100] Ravikumar R. Steiner A. Assaad F. F. (2017). Multisubunit tethering complexes in higher plants. Curr. Opin. Plant Biol. 40, 97–105. doi: 10.1016/j.pbi.2017.08.009 28889036

[B101] Reichardt I. Slane D. El Kasmi F. Knoll C. Fuchs R. Mayer U. . (2011). Mechanisms of functional specificity among plasma-membrane syntaxins in *Arabidopsis* . Traffic 12, 1269–1280. doi: 10.1111/j.1600-0854.2011.01222.x 21707889

[B102] Renna L. Stefano G. Slabaugh E. Wormsbaecher C. Sulpizio A. Zienkiewicz K. . (2018). TGNap1 is required for microtubule-dependent homeostasis of a subpopulation of the plant *trans*-golgi network. Nat. Commun. 9, 5313. doi: 10.1038/s41467-018-07662-4 30552321 PMC6294250

[B103] Richter S. Kientz M. Brumm S. Nielsen M. E. Park M. Gavidia R. . (2014). Delivery of endocytosed proteins to the cell-division plane requires change of pathway from recycling to secretion. Elife 3, e02131. doi: 10.7554/eLife.02131 24714496 PMC3979144

[B104] Risselada H. J. Mayer A. (2020). SNAREs, tethers and SM proteins: how to overcome the final barriers to membrane fusion? Biochem. J. 477, 243–258. doi: 10.1042/BCJ20190050 31951000

[B105] Rodriguez-Furlan C. Minina E. A. Hicks G. R. (2019). Remove, recycle, degrade: regulating plasma membrane protein accumulation. Plant Cell 31, 2833–2854. doi: 10.1105/tpc.19.00433 31628169 PMC6925004

[B106] Rosquete M. R. Worden N. Ren G. Sinclair R. M. Pfleger S. Salemi M. . (2019). AtTRAPPC11/ROG2: a role for TRAPPs in maintenance of the plant *trans*-golgi network/early endosome organization and function. Plant Cell 31, 1879–1898. doi: 10.1105/tpc.19.00110 31175171 PMC6713296

[B107] Rubiato H. M. Liu M. O'Connell R. J. Nielsen M. E. (2022). Plant SYP12 syntaxins mediate an evolutionarily conserved general immunity to filamentous pathogens. ELife 11, e73487. doi: 10.7554/eLife.73487 35119361 PMC8865848

[B108] Rui Q. Tan X. Liu F. Li Y. Liu X. Li B. . (2021). Syntaxin of plants31 (SYP31) and SYP32 is essential for golgi morphology maintenance and pollen development. Plant Physiol. 186, 330–343. doi: 10.1093/plphys/kiab049 33576796 PMC8154079

[B109] Rybak K. Steiner A. Synek L. Klaeger S. Kulich I. Facher E. . (2014). Plant cytokinesis is orchestrated by the sequential action of the TRAPPII and exocyst tethering complexes. Dev. Cell 29, 607–620. doi: 10.1016/j.devcel.2014.04.029 24882377

[B110] Saito C. Ueda T. (2009). Chapter 4: functions of RAB and SNARE proteins in plant life. Int. Rev. Cell Mol. Biol. 274, 183–233. doi: 10.1016/s1937-6448(08)02004-2 19349038

[B111] Samuels A. L. Giddings T. H. Jr. Staehelin L. A. (1995). Cytokinesis in *tobacco* BY-2 and root tip cells: a new model of cell plate formation in higher plants. J. Cell Biol. 130, 1345–1357. doi: 10.1105/tpc.106.040923 7559757 PMC2120572

[B112] Sanderfoot A. (2007). Increases in the number of SNARE genes parallels the rise of multicellularity among the green plants. Plant Physiol. 144, 6–17. doi: 10.1104/pp.106.092973 17369437 PMC1913785

[B113] Seguí-Simarro J. M. Austin J. R. White E. A. Staehelin L. A. (2004). Electron tomographic analysis of somatic cell plate formation in meristematic cells of *Arabidopsis* preserved by high-pressure freezing. Plant Cell 16, 836–856. doi: 10.1105/tpc.017749 15020749 PMC412860

[B114] Sinclair R. Hsu G. Davis D. Chang M. Rosquete M. Iwasa J. H. . (2022). Plant cytokinesis and the construction of new cell wall. FEBS Lett. 596, 2243–2255. doi: 10.1002/18733468.14426 35695093

[B115] Smertenko A. Assaad F. Baluska F. Bezanilla M. Buschmann H. Drakakaki G. . (2017). Plant cytokinesis: terminology for structures and processes. Trends Cell Biol. 27, 885–894. doi: 10.1016/j.tcb.2017.08.008 28943203

[B116] Sohn E. J. Kim E. S. Zhao M. Kim S. J. Kim H. Kim Y. W. . (2003). Rha1, an *Arabidopsis* Rab5 homolog, plays a critical role in the vacuolar trafficking of soluble cargo proteins. Plant Cell 15, 1057–1070. doi: 10.1105/tpc.009779 12724533 PMC153716

[B117] Söllner R. Glässer G. Wanner G. Somerville C. R. Jürgens G. Assaad F. F. (2002). Cytokinesis-defective mutants of *Arabidopsis* . Plant Physiol. 129, 678–690. doi: 10.1104/pp.004184 12068111 PMC161693

[B118] Speth E. B. Imboden L. Hauck P. He S. Y. (2009). Subcellular localization and functional analysis of the *Arabidopsis* GTPase RabE. Plant Physiol. 149, 1824–1837. doi: 10.1104/pp.108.132092 19233904 PMC2663744

[B119] Stenmark H. (2009). Rab GTPases as coordinators of vesicle traffic. Nat. Rev. Mol. Cell Biol. 10, 513–525. doi: 10.1038/nrm2728 19603039

[B120] Stierhof Y. D. El Kasmi F. (2010). Strategies to improve the antigenicity, ultrastructure preservation and visibility of trafficking compartments in *Arabidopsis* tissue. Eur. J. Cell Biol. 89, 285–297. doi: 10.1016/j.ejcb.2009.12.003 20106548

[B121] Südhof T. C. Rothman J. E. (2009). Membrane fusion: grappling with SNARE and SM proteins. Science 323, 474–477. doi: 10.1126/science.1161748 19164740 PMC3736821

[B122] Suwastika I. N. Uemura T. Shiina T. Sato M. H. Takeyasu K. (2008). SYP71, a plant-specific qc-SNARE protein, reveals dual localization to the plasma membrane and the endoplasmic reticulum in *Arabidopsis* . Cell Struct. Funct. 33, 185–192. doi: 10.1247/csf.08024 18827404

[B123] Synek L. Pleskot R. Sekeres J. Serrano N. Vukasinovic N. Ortmannova J. . (2021). Plasma membrane phospholipid signature recruits the plant exocyst complex *via* the EXO70A1 subunit. Proc. Natl. Acad. Sci. U. S. A. 118, e2105287118. doi: 10.1073/pnas.2105287118 34470819 PMC8433549

[B124] Synek L. Schlager N. Elias M. Quentin M. Hauser M. T. Zarsky V. (2006). AtEXO70A1, a member of a family of putative exocyst subunits specifically expanded in land plants, is important for polar growth and plant development. Plant J. 48, 54–72. doi: 10.1111/j.1365-313X.2006.02854.x 16942608 PMC2865999

[B125] Takáč T. Pechan T. Samajová O. Ovečka M. Richter H. Eck C. . (2012). Wortmannin treatment induces changes in *Arabidopsis* root proteome and post-golgi compartments. J. Proteome. Res. 11, 3127–3142. doi: 10.1021/pr201111n 22524784

[B126] Takemoto K. Ebine K. Askani J. C. Kruger F. Gonzalez Z. A. Ito E. . (2018). Distinct sets of tethering complexes, SNARE complexes, and rab GTPases mediate membrane fusion at the vacuole in *Arabidopsis* . Proc. Natl. Acad. Sci. U.S.A. 115, E2457–E2466. doi: 10.1073/pnas.1717839115 29463724 PMC5877921

[B127] Tan X. Feng Y. Liu Y. Bao Y. (2016). Mutations in exocyst complex subunit SEC6 gene impaired polar auxin transport and PIN protein recycling in *Arabidopsis* primary root. Plant Sci. 250, 97–104. doi: 10.1016/j.plantsci.2016.06.001 27457987

[B128] Tan X. Xu H. Ye J. Wang J. Liu W. Liu F. . (2022). *Arabidopsis* exocyst subunit SEC6 is involved in cell plate formation during microgametogenesis. Biochem. Biophys. Res. Commun. 598, 100–106. doi: 10.1016/j.bbrc.2022.01.092 35151976

[B129] Thellmann M. Rybak K. Thiele K. Wanner G. Assaad F. F. (2010). Tethering factors required for cytokinesis in *Arabidopsis* . Plant Physiol. 154, 720–732. doi: 10.1104/pp.110.154286 20713617 PMC2948999

[B130] Tulin F. Cross F. R. (2014). A microbial avenue to cell cycle control in the plant superkingdom. Plant Cell 26, 4019–4038. doi: 10.1105/tpc.114.129312 25336509 PMC4247570

[B131] Vernoud V. Horton A. C. Yang Z. Nielsen E. (2003). Analysis of the small GTPase gene superfamily of *Arabidopsis* . Plant Physiol. 131, 1191–1208. doi: 10.1104/pp.013052 12644670 PMC166880

[B132] Völker A. Stierhof Y. D. Jürgens G. (2001). Cell cycle-independent expression of the *Arabidopsis* cytokinesis-specific syntaxin KNOLLE results in mistargeting to the plasma membrane and is not sufficient for cytokinesis. J. Cell Sci. 114, 3001–3012. doi: 10.1242/jcs.114.16.3001 11686303

[B133] Vukašinović N. Cvrčková F. Eliáš M. Cole R. Fowler J. E. Žárský V. . (2014). Dissecting a hidden gene duplication: the *Arabidopsis thaliana* SEC10 locus. PloS One 9, e94077. doi: 10.1371/journal.pone.0094077 24728280 PMC3984084

[B134] Vukašinović N. Žárský V. (2016). Tethering complexes in the arabidopsis endomembrane system. Front. Cell. Dev. Biol. 4, 46. doi: 10.3389/fcell.2016.00046 27243010 PMC4871884

[B135] Waizenegger I. Lukowitz W. Assaad F. Schwarz H. Jürgens G. Mayer U. (2000). The *Arabidopsis* KNOLLE and KEULE genes interact to promote vesicle fusion during cytokinesis. Curr. Biol. 10, 1371–1374. doi: 10.1016/s0960-9822(00)00775-2 11084337

[B136] Wickner W. Schekman R. (2008). Membrane fusion. Nat. Struct. Mol. Biol. 15, 658–664. doi: 10.1038/nsmb.145 18618939 PMC2488960

[B137] Won K. H. Kim H. (2020). Functions of the plant qbc SNARE SNAP25 in cytokinesis and biotic and abiotic stress responses. Mol. Cells 43, 313–322. doi: 10.14348/molcells.2020.2245 32274918 PMC7191049

[B138] Woollard A. A. Moore I. (2008). The functions of rab GTPases in plant membrane traffic. Curr. Opin. Plant Biol. 11, 610–619. doi: 10.1016/j.pbi.2008.09.010 18952493

[B139] Wu J. Tan X. Wu C. Cao K. Li Y. Bao Y. (2013). Regulation of cytokinesis by exocyst subunit SEC6 and KEULE in *Arabidopsis thaliana* . Mol. Plant 6, 1863–1876. doi: 10.1093/mp/sst082 23702595

[B140] Xia L. Mar Marques-Bueno M. Bruce C. G. Karnik R. (2019). Unusual roles of secretory SNARE SYP132 in plasma membrane h^+^-ATPase traffic and vegetative plant growth. Plant Physiol. 180, 837–858. doi: 10.1104/pp.19.00266 30926657 PMC6548232

[B141] Yi P. Goshima G. (2022). Division site determination during asymmetric cell division in plants. Plant Cell 34, 2120–2139. doi: 10.1093/plcell/koac069 35201345 PMC9134084

[B142] Yu I. M. Hughson F. M. (2010). Tethering factors as organizers of intracellular vesicular traffic. Annu. Rev. Cell Dev. Biol. 26, 137–156. doi: 10.1146/annurev.cellbio.042308.113327 19575650

[B143] Yun H. S. Kwaaitaal M. Kato N. Yi C. Park S. Sato M. H. . (2013). Requirement of vesicle-associated membrane protein 721 and 722 for sustained growth during immune responses in *Arabidopsis* . Mol. Cells 35, 481–488. doi: 10.1007/s10059-013-2130-2 23661365 PMC3887875

[B144] Zhang J. Chen J. Wang L. Zhao S. Li J. Liu B. . (2018). AtBET5 is essential for exine pattern formation and apical meristem organization in *Arabidopsis* . Plant Sci. 274, 231–241. doi: 10.1016/j.plantsci.2018.05.033 30080609

[B145] Zhang Z. Feechan A. Pedersen C. Newman M. A. Qiu J. L. Olesen K. L. . (2007). A SNARE-protein has opposing functions in penetration resistance and defence signalling pathways. Plant J. 49, 302–312. doi: 10.1111/j.1365-313X.2006.02961.x 17241452

[B146] Zhang Y. Immink R. Liu C. M. Emons A. M. Ketelaar T. (2013). The *Arabidopsis* exocyst subunit SEC3A is essential for embryo development and accumulates in transient puncta at the plasma membrane. New Phytol. 199, 74–88. doi: 10.1111/nph.12236 23495664

[B147] Zhang L. Ma J. Liu H. Yi Q. Wang Y. Xing J. . (2021). SNARE proteins VAMP721 and VAMP722 mediate the post-golgi trafficking required for auxin-mediated development in *Arabidopsis* . Plant J. 108, 426–440. doi: 10.1111/tpj.15450 34343378

[B148] Zhang B. Wang H. Zhang Y. (2020). SNARE proteins and their role in plant ion channel regulation. Plant Growth Regul. 92, 443–453. doi: 10.1007/s10725-020-00656-7

[B149] Zhang L. Zhang H. Liu P. Hao H. Jin J. B. Lin J. (2011). *Arabidopsis* r-SNARE proteins VAMP721 and VAMP722 are required for cell plate formation. PloS One 6, e26129. doi: 10.1371/journal.pone.0026129 22022536 PMC3191180

[B150] Zheng H. Bednarek S. Y. Sanderfoot A. A. Alonso J. Ecker J. R. Raikhel N. V. (2002). NPSN11 is a cell plate-associated SNARE protein that interacts with the syntaxin KNOLLE. Plant Physiol. 129, 530–539. doi: 10.1104/pp.003970 12068098 PMC161670

[B151] Zheng H. Camacho L. Wee E. Batoko H. Legen J. Leaver C. J. . (2005). A rab-e GTPase mutant acts downstream of the rab-d subclass in biosynthetic membrane traffic to the plasma membrane in *tobacco* leaf epidermis. Plant Cell 17, 2020–2036. doi: 10.1105/tpc.105.031112 15972698 PMC1167549

[B152] Zmienko A. Marszalek-Zenczak M. Wojciechowski P. Samelak-Czajka A. Luczak M. Kozlowski P. . (2020). AthCNV: a map of DNA copy number variations in the *Arabidopsis* genome. Plant Cell 32, 1797–1819. doi: 10.1105/tpc.19.00640 32265262 PMC7268809

